# Round the Hospitals

**Published:** 1916-06-24

**Authors:** 


*(To be continued.)*

### ROUND THE HOSPITALS.

The meeting at St. Thomas's Hospital in support of the College of Nursing on the 15th instant was one of the largest ever held in the Great Hall of that institution. It was summoned by Mr. Stanley as a meeting of nominated representatives of the nurse-training schools of hospitals and Poor-Law infirmaries throughout the country. An examination of the names of those present, however, shows that some fifty other persons obtained admission and may have voted at the Chairman's invitation, though they were not representatives or accredited to the meeting. Care will no doubt be taken in future to provide that only nominated representatives will obtain admission to further conferences of the kind. If in Mr. Stanley's letter of the 7th instant a card had been included printed on the back with a space for the name of the institution, and lines for signatures and titles of the representatives appointed, with an intimation that it must be presented at the door to obtain admission to the meeting, unaccredited persons would not have been admitted. We give on page 307 a full report of the proceedings at the meeting, and confess to the feeling that the majority, however constituted, were right in supporting the proposal that the Consultative Board of the College should as a whole be summoned to deal with the matters submitted to them by the Council for their advice and recommendations. The Consultative Board can readily adjourn when necessary to enable matters of technical importance to be referred to small and competent committees with instructions to prepare a draft memorandum or report for the future consideration of the Board, and, subject to its approval, for submission to the Council in due course.

The thoughts of matrons are now busily occupied with arrangements for summer holidays. The problem of providing extra help for holiday duties must be settled either by temporarily promoting nurses to sisters' duties or engaging holiday sisters. Every hospital has its own special needs, and often there is a reason for not promoting nurses already at work in the wards; they may not be sufficiently reliable or the admission of extra probationers to fill the gaps in the ranks made by promotion may render the staff too large when the holidays are over. There is, however, no doubt that the privilege of undertaking sisters' duties for three or four weeks is greatly valued by nurses who are almost qualified, and the plunge into such responsible work is more easily made in a ward with which the nurse is familiar than in a new hospital where everything is strange and unaccustomed. An acting sister undertaking these duties for the first time should realise that the matron is at hand in any difficulty to give advice, and she will be grateful for occasional visits to the ward made during the day by the matron. The sister will thus have an opportunity to show when all goes well how orderly and methodical the ward is, and in case of difficulty she will feel that it is not even necessary to go and find a counsellor, her matron is there ready to help.

---

One problem which has exercised many minds in the nursing world is exactly what effect the war will be found to have upon probationers whose period of training took place in war time, and if similar causes will affect, and if so to what extent, other nurses who were undergoing their training at the commencement of the war. Speaking generally, the facts include cases where vacancies in the post of sister have been filled by selections from the staff nurses who remained at the civil hospitals, where it was found essential sometimes to promote a staff nurse to act as sister at the end of her third year. It is even hinted that in some cases matrons were obliged to promote staff nurses of less than two years' training. The efforts to meet the special emergencies just referred to must influence the efficiency and knowledge of many staff nurses, and consequently the efficiency of the work done in some civil hospitals during war time. The effect upon the charge nurse and her future career and knowledge cannot fail to handicap some, at any rate, of those promoted to be sisters, owing to the fact that several of them may have lost their third year, a most valuable part of every nurse's training, or that it may have been deprived of much of its value owing to the absence of an experienced sister under whom in ordinary times she would have had the privilege of working. Further, the promotion of a charge nurse with less than three years' training must deprive the ward in which she is acting as sister of knowledgeable supervision, and when the staff nurse is conscientious the effect of such a position upon herself may be to create harass and worry. But the mischief does not end even here, for in those hospitals which have been admitting probationers during war time no adequate system of training can be possible where ward sisters are without adequate experience and complete training in ward management and the higher duties of a nurse. Hence both the staff nurses who have been hurriedly promoted and the probationers who may have joined the hospital for training may suffer grievously and permanently.

---

It is a grave question whether the larger hospitals which have experienced difficulties of the kind just referred to can, in justice to their deservedly high reputation, continue to take probationers for training in such circumstances during war time. A possible remedy would be for the matron to appoint a few experienced, knowledgeable, and competent ward sisters whom she could place in temporary charge of the wards to which charge nurses had had to be hurriedly promoted, so that the latter might have adequate and continuous training in ward management of the best. We should welcome a discussion, an account of the difficulties already experienced, and any suggestions as to the best way of overcoming them. War time is the parent of increasing difficulties to nearly everybody in authority, and an exchange of experiences could not fail to prove helpful and possibly to promote an efficient remedy.

---

We have not space to give a full report of the meeting of the Cottage Benefit Nursing Association, which was held on the 14th instant, a full report of which will be found in The Nursing Mirror of the 24th instant. This Association has done some good work, and its course has led to regrettable happenings. The system has yet to be perfected, if this is capable of accomplishment. It is held by many able workers in the nursing field that the present system rests on wrong bases—*i.e.*, imperfect training, and an absence of adequate sleeping accommodation. It is essential to point out these facts because the afternoon meeting really became an occasion for abuse of the College of Nursing, with which—initially, at any rate—the Cottage Benefit Nursing Association can have little or no concern; that is to say, the College of Nursing has first of all to provide for trained nurses and to overcome many difficulties which surround this essential provision before they can extend their work to similar organisations affecting various groups of so-called nurses with training of various kinds, though some of it may be altogether short of that received by certificated trained nurses. We venture to think that Miss Broadwood and Miss Joseph would have been well advised to have exhibited a little patience. They would then have deferred such remarks as they had to make for a later occasion when the College has been organised and its work commenced. We are in full sympathy with an adequate scheme for the provision of trained nurses in the villages of the United Kingdom. Our point is that, although there must invariably be a number of different types or classes of nurses, if the whole population is to be adequately provided for in sickness the College of Nursing's first duty is to set up a Register and include in it certificated trained nurses of the required standard before proceeding to deal with other grades of which the village or the cottage nurse on approved conditions may ultimately prove to be one.

For Matrons' and Sisters' Appointments see page 286.

